# Binomial names for virus species: the rediscovery of an old idea

**DOI:** 10.1099/jgv.0.002102

**Published:** 2025-06-09

**Authors:** Stuart G. Siddell, Donald B. Smith

**Affiliations:** 1 School of Cellular and Molecular Medicine, University of Bristol, Bristol, UK; 2 Nuffield Department of Medicine, University of Oxford, Oxford, UK

**Keywords:** binomial species names, history, virus taxonomy

## Abstract

The International Committee on Taxonomy of Viruses now mandates that all virus species names be presented in a binomial format. This requirement replaces the various naming formats that have been used since the first official virus taxonomy Report was published in 1971. A review of virus classification schemes as they have developed over the past century shows that, although there was an initial inclination to adopt a Linnaean binomial nomenclature, various other naming formats were gradually introduced for practical and scientific reasons. However, as our understanding of viruses has advanced – especially with the increasing availability of genomic sequences – the arguments for these alternative formats (such as that viruses were not living or that they evolved too quickly) have diminished. The nomenclature for virus species now aligns more closely with the conventions used in other areas of biology, the format having nearly come full circle from its beginnings a century ago.

## Introduction

Virus classification is concerned with developing a hierarchal scheme of conceptual units (called taxa) that, ideally, mirrors the relationships between viruses allocated to these taxa. A separate but closely related process involves giving these taxa names. Decisions about how viruses should be allocated to taxa are often complex, sometimes relying on new scientific discoveries or requiring judgements on the relative importance of genetic, structural and biological properties. In recent decades, there has been an additional challenge arising from the volume of virus sequence information generated by high-throughput sequencing of metagenomic samples. Such studies reveal a huge diversity of viruses in the environment but often do not provide any information on phenotypic attributes, such as virion morphology, host range, epidemiology or pathogenicity. Despite the increasing complexity of the different approaches used in virus classification, it has often been the taxon naming step, particularly the naming of the species taxon, that has produced the most controversy.

During the initial phase of discovery, viruses were generally referred to by the disease that they caused [1,3]. For example, Loeffler and Frosch refer to the ‘agent of foot-and-mouth disease’ (‘*Erregers der Maul und Klauenseuche*’) [3]. This practice changed in the 1930s when the physico-chemical properties and morphology of virus particles were investigated, and the modern concept of a virus began to emerge. Thus, for example, from this time onwards, the agent of tobacco mosaic disease was referred to mostly as tobacco mosaic virus [4]. Common names such as these are still used for viruses, and they are largely decided upon by the virologists who study them. This virus naming process is not regulated, and the International Committee on Taxonomy of Viruses (ICTV) has no official role.

In contrast, the ICTV is mandated to develop an internationally agreed-upon taxonomy for viruses, to establish internationally agreed-upon names for virus taxa and to maintain an official index of approved names for virus taxa (ictv.global/about/statutes). This index is provided on the ICTV website (ictv.global); both visually (Virus Taxonomy Browser) and as a spreadsheet [Master Species List (MSL)], both including links to the history of each taxon. An additional spreadsheet gives exemplar viruses for each species (Virus Metadata Resource). Since its inception, the ICTV has published reports (as articles or books, and now online) providing contemporaneous taxonomic information as well as describing the properties and characteristics of the members of each taxon (Table 1).

**Table 1.** ICNV/ICTV Reports on the classification and nomenclature of viruses

Year	Report	Reference/editor	MSL release
1971	ICNV First Report	[33]	MSL#01
1976	ICTV Second Report	[34]	MSL#04
1979	ICTV Third Report	[35]	MSL#06
1982	ICTV Fourth Report	[36]	MSL#08
1991	ICTV Fifth Report	[37]	MSL#12
1995	ICTV Sixth Report	[38]	MSL#14
2000	ICTV Seventh Report	[39]	MSL#18
2005	ICTV Eighth Report	[40]	MSL#23
2012	ICTV Ninth Report	[41]	MSL#25
2017–2018	ICTV Online Report	E.J. Lefkowitz*	MSL#32–33
2018–2024	ICTV Online Report	S.G. Siddell*	MSL#34–39
2024–now	ICTV Online Report	E.M. Adriaenssens*	MSL#40–now

*The Online Report is available at https://ictv.global/report.

In this short article, we aim to review the development of virus species nomenclature (i.e. the naming of virus species taxa) over the last century, particularly the use of binomial species names. This history reveals that although virus species were first named using Linnaean binomials, as elsewhere in biology (Box 1), by the 1960s their continued use was judged to be inappropriate or premature. Binomial virus species names were ignored and supplanted by virus species names in a multitude of different typographies and orthographies. The current ICTV position on virus species names (mandated binomial names with either Latinized or non-Latinized species epithets) can be viewed as the latest stage in a process that now aligns virus species nomenclature much more closely with the rest of biology.

Box 1. **Binomial nomenclature**

Carl Linnaeus (1707–1778) formalized binomial nomenclature. The binomial naming system uses just two words, the genus name and the species name (or species epithet), to uniquely identify biological species. Linnaeus’ major contribution was to use a single word as the species epithet, avoiding the use of descriptive polynomials that had become longer and longer as the number of species in a genus increased.

Linnaeus proposed that the species epithet could be chosen from any source (although they are often derived from the names of people or places), including new words. He even referred to them as *nomina trivialia* (trivial names), which allowed for arbitrary names. The binomial names, or scientific names, that Linnaeus used have Latin grammatical forms or were words of another language converted to Latin. However, the current ICTV code allows species epithets to be freeform, i.e. non-Latinized, although guidance is also available for the creation of Latinized epithets [26].

The use of Linnaean binomial scientific names provides concise, uniform and indubitable names that convey taxonomic information and are recognized internationally, unlike common (or vernacular) names. The binomial system is not perfect, but the longevity of the system attests to its usefulness.

## Virus classification

Virus classification shapes the practice of virus nomenclature. Beginning in the 1920s, the first tentative discussions of how to classify plant viruses encompassed various alternatives, ranging from numerical [5] or binomial [6] naming systems based on the host to those based on the symptoms produced in infected hosts [7]. A notable first attempt to produce a classification encompassing all known viruses was in 1948 by Holmes [8], extending his earlier work on plant viruses. This system used a Linnaean binomial nomenclature and was published as an appendix to the sixth edition of Bergey’s Manual of Determinative Bacteriology.

Despite Holmes’ impressive efforts in describing 248 virus species in 32 genera, 13 families and 3 suborders, this classification scheme did not meet with universal approval. This was not because of the use of Linnaean binomial names, but because of the general nature of the features that were used to group different taxa. For example, the three suborders in the order *Virales* were established based purely on whether the host was bacterial (*Phagineae*), plant (*Phytophagineae*) or animal (*Zoophagineae*). Genera and species were distinguished by characteristics such as host specificity, disease symptoms, vector, inactivation by heat, particle size and plaque size.

Nevertheless, some virologists saw the Holmes classification as a useful starting point from which an improved system of classification could be produced. For example, considering just the insect viruses, in 1949, Steinhaus [9] proposed two additional genera, reframing the genus definition to reflect the type of inclusion bodies produced rather than the host order, moved species between genera and established additional species. Another system, developed in the 1960s, also adopted a binomial nomenclature but based upon different characters from those used by Holmes [10,11]. In this system, known (from the first letters of the authors’ surnames, Lwoff, Horne and Tournier) as LHT, the emphasis was placed on virus characters such as the nature of the genetic material, the symmetry of the capsid and the naked or enveloped nature of the nucleocapsid. This approach resulted in groups that were arranged from phyla to species. The footprint of LHT can still be seen in the current ICTV nomenclature, for example, in the names of the families *Poxviridae*, *Adenoviridae* and *Paramyxoviridae* and in the suffixes *-viral*es, *-viridae* and -*virinae* used for taxa of different ranks.

However, not all virologists of the period approved, arguing that the Holmes and LHT classifications were premature and unhelpful because the genera did not comprise groups of similar viruses: ‘We feel that there is no value in introducing at present a full Linnaean nomenclatural system because current ideas on virus classification are insufficiently mature’ [12]. The objection was more to the perceived inadequate scientific basis for these classification schemes, rather than their use of a binomial nomenclature.

An alternative system proposed in 1968 was based on cryptograms, these being short summaries of virus properties, followed by a group name, of the form ‘D/2 : 74/* : S/S : V/O, Herpes-group’ [12], and, in this case, encoding the information D: DNA genome; 2: double-stranded genome; 74: genome size (in MDaltons); S/S: virion outline and particle that are spherical; V: vertebrate host range; and O: non-vectored spread. Similarly, it was suggested that binomial names could be improved if they were extended with additional terms ‘in order to mark the place of the virus in the hierarchy’, for example, Papillomavirus sylvilagus (Dc, H), where Dc indicates a DNA genome within a cubical capsid and H indicates the absence of a lipid envelope [13].

Further objections made during the 1950s and 1960s to the use of Latinized binomial names were that they might not be appropriate for organisms that were not living [14] or did not reproduce sexually [15] or evolved too quickly [14]. It was also suggested that the use of Latinized binomial names implied phylogenetic relationships that were as yet unknown [16] and would be divisive if not introduced simultaneously for both animal and plant viruses [16], that Latin was no longer widely understood and that a second set of names would have to be learnt by virologists. More flippantly, but revealing a lot about the tensions surrounding the issue, it was remarked ‘that [the] main outcome of adopting the LB [Latinized Binomial] system may be that virus taxonomists are kept happy and occupied’ [15].

After the establishment of the ICTV in 1966 (see below) and the tentative development of an ‘official’ taxonomy and associated nomenclature, alternative classification schemes were described. For example, a major contribution was the system developed in 1971 by Baltimore [17], which was based upon genome type and the relation of the genome to the synthesis of mRNA. This system became, and still is, widely used, possibly because it was easy to understand and all-inclusive. Since then, classifications have been proposed to collect viruses into high-level groups, for example, the alphavirus-like and picornavirus-like superfamilies of positive-strand RNA viruses [18,19] and cytoplasmic DNA viruses [20]. These higher-level classifications had no significant effect on the ‘official’ ICTV taxonomy and nomenclature at the species level. However, traces of them can be seen in the current nomenclature of some higher rank taxa; for example, the phylum *Pisuviricota* is analogous to the picornavirus supergroup.

The recent era of virus classification has been affected by the introduction of rapid and inexpensive high-throughput sequencing. Notably, there has been a shift away from a classification based on disease or phenotypic properties and towards one based on genetic relationships. These relationships can be explored in a variety of ways. For example, the comparison of nucleotide or protein sequences may involve multiple sequence alignment, a pairwise (or patristic) distance matrix, virus clustering [21,22] and phylogenetic reconstructions of homologous virus genes to infer evolutionary relationships. Alternatively, there are approaches that involve so-called ‘genome organizational models’, which identify the protein homology content of virus genomes and then cluster viruses based on gene synteny [23,24]. A recent development has been the classification of distantly related viruses based on a comparison of conserved secondary structure domains within structural proteins [25]. The results of all these comparative analyses are often depicted in the form of phylogenetic trees or dendrograms that can demonstrate the monophyly of the classified groups.

Again, these new approaches to virus classification do not, per se, necessitate nomenclatural changes. However, it is clear that with the introduction of sequence-based classification, many more taxa will need to be named throughout the taxonomic hierarchy. In relation to the binomial naming of species taxa, novel approaches to the computer-assisted generation of large batches of genus and species names have already been devised [26] and implemented [27]. Some examples of the resulting binomial species names (*Glyciruvirus geovivens* and *Kecuhnavirus borborohabitans*) show that this type of approach can produce sensible, informative and relatively simple names.

## International initiatives to co-ordinate virus taxonomy

Before, and leading up to the establishment of the ICTV, several attempts were made to develop international agreements about how viruses could be classified and named in a unified system. First, plant virologists made attempts at the fifth (1930) and sixth (1935) International Botanical Congress meetings to establish a committee and then agree on a system of nomenclature [28]. A tentative agreement was reached to follow the system of Johnson [5], later extended by Smith [29], and a 32-page mimeographed prospectus was produced. It was hoped to reach an agreement with other virologists at the International Microbiology Congress in New York in September 1939; however, war was declared in Europe on the opening day of this congress, and progress came to an end. Similarly, a committee of the American Phytopathological Society was set up in 1938 but was unable to reach an agreement on a system of nomenclature [28].

A second attempt focussed on a meeting in 1952 at the New York Academy of Sciences, which resulted in a monograph forming volume 56 of the Annals of the New York Academy of Sciences. In an introduction to this volume, Burnet wrote that it was important to ‘go all out to make a start on virus classification’ and ‘… nor does it seem necessary to look beyond the Linnean binomial system for the form of the names that might eventually be suggested’ [30]. However, Burnet also recognized that there were many virologists of a different opinion, and, at least for animal viruses, he later wrote: ‘The system adopted can now be regarded as one of convenient and unpretentious binomials; the group names being a combination of an appropriate prefix with “virus” as Poxvirus, Poliovirus, and so on.’ [31]. This convenience must not have been apparent, as, within a few years, the situation was that ‘the names Myxovirus and Poliovirus have been widely used, but the “specific” names have been practically ignored … there is little enthusiasm or demand for Latin names for individual animal viruses.’ [32].

A third, and ultimately more enduring, international effort began at the International Congress of Microbiology held in Rio de Janeiro in 1950. Prior to the meeting, CH Andrewes had been invited to form a committee to explore the field of virus taxonomy and present a Report. Unfortunately, at the congress itself, it was concluded that (1) the starting date for a valid virus nomenclature should be fixed at a later congress and (2) that the use of comprehensive systems of nomenclature and classification of viruses at the present moment was unwise [14]. However, virologists continued to deliberate between subsequent congresses (Rome, 1953; Stockholm, 1958; Montreal, 1962), and by 1964, another committee had been formed (the Provisional Committee on Nomenclature of Viruses, PCNV) but could agree to only three over-riding resolutions: that the bacteriological code should not be applied to viruses, that virus nomenclature should be internationally adopted and that virus nomenclature should be applied to all viruses. It was in this frame of mind that the PCNV was dissolved, and an enlarged International Committee on Nomenclature of Viruses (ICNV) was founded and began its deliberations at the ninth International Congress of Microbiology in Moscow in 1966.

## Evolution of ICTV policies

In 1966, the ICNV established four specialist subcommittees (vertebrate virus, plant virus, invertebrate virus and bacteriophage) that were asked to suggest classification schemes and nomenclature in their area of expertise. The results of these efforts were summarized in the first Report of the ICNV [33]. The most important decisions made at that time were to (1) define groups of viruses with a short description of the main discriminating characteristics, (2) designate some groups as families or genera, (3) provide each family or genus with a type member (genus or species) and (4) list members, probable members and possible members for each family and genus. A limited effort was made towards a Latinized binomial nomenclature, although the use of numbers and letters was also accepted for species names.

It was clear from the beginning that a binomial species name format did not find uniform approval with the various ICTV subcommittees and study groups (the ICNV was renamed ICTV in 1975). Early reports listed what were described as ‘Current’ or ‘Vernacular’ names, these being identical with virus names, but grouped under the title of ‘Other species’. A set of binomial species names suggested by the ICTV was not adopted, and there were no species name entries in the column for ‘Approved name’ in the first [33] and second reports [34] or under ‘International name’ (presumably used as meaning the approved, scientific name) in the third [35], fourth [36] and fifth [37] reports. This uncertainty in naming virus species taxa is also apparent in a discussion of the issues by REF Matthews in the third ICTV Report, where species are considered as being a category between that of genus and varieties, yet distinguished typographically as italicized, with binomial names such as ‘*Enterovirus polio 1*’ and ‘*Enterovirus 70*’ beginning with the genus name, although not always being binomial.

In the fourth ICTV Report [36], the rules of nomenclature were revised to include several new rules: 12 – ‘The genus name and species epithet, together with the strain designation, must give an unambiguous identification of the virus’, 13 – ‘The species epithet must follow the genus name and be placed before the designation of strain, variant or serotype’ and 14 – ‘A species epithet should consist of a single word, or if essential, a hyphenated word. The word may be followed by numbers or letters’. These rules seemed to represent a more unified approach to nomenclature and, at least, some acceptance of binomial names.

Then, in an unexplained and confusing series of changes, the fifth ICTV Report [37] abolished Rule 13 (genus name followed by species epithet) and Rule 4 which stated that an effort should be made towards a Latinized nomenclature. New Rules 11 and 12 refer to ‘virus names’ rather than ‘species epithets’. One interpretation of this is that in moving away from a binomial format, the term ‘species epithet’ was no longer appropriate since it would usually be used as a term to follow a genus name, and so the amended rules may be treating ‘virus name’ as equivalent to ‘species name’. At any rate, the only names given for the type species of a genus are labelled ‘English vernacular name’ and are written in capital letters of plain text and often followed by a strain or serotype designation, e.g. HUMAN (ALPHA) HERPESVIRUS 1 (HERPES SIMPLEX VIRUS 1). In a further departure from binomial orthography, the sixth ICTV Report [38] stated that ‘species designations’ (species names) should not be italicized or capitalized unless derived from a place name or host family or genus. Since, in many cases, the lists of virus species corresponded largely with the viruses listed as genus members in earlier reports, the distinction between viruses and virus species was thereby blurred both conceptually and typographically.

Then, just a few years later in the seventh Report [39], this rule was reversed so that species names were now stipulated to be comprised of as few words as practicable and to be italicized with the first word capitalized. The detailed conceptual explanation for these changes was provided in a section of the Report written by Marc H. V. van Regenmortel. The result was that many of the more than 1,600 species documented in the seventh Report had species names identical, except typographically, to virus names. This practice continued with the eighth [40] and ninth [41] reports, although a significant number of species names were of an inverted binomial or polynomial construction with a species epithet of one or more words followed by the genus name as a lowercase word. The stated advantage of this format was that it indicated the genus to which a species belonged and that placing the genus name after the current species name ensured that the name ended in ‘virus’ [42]. Unfortunately, this approach was not helped by the lack of a rule that required all virus species to be placed in a genus, a situation that was not addressed until 2018 (taxonomy proposal 2017.005G.A.v1.Additional Taxonomy Ranks).

In any case, de facto, the typographical distinction between virus names and virus species names proved insufficient to consistently discriminate between them, and this was part of the motivation for several years of discussion on the topic by the ICTV Executive Committee. Following wider consultation [43] and the ratification in 2021 of the taxonomy proposal 2018.001 G.R.binomial_species, virus species names are now mandated to be binomial with a genus name that ends with ‘*virus*’ followed by a one-word species epithet, either in a Latinized form (e.g. *Orthoebolavirus zairense*) or freeform (e.g. *Simplexvirus humanalpha1*). The only limitation to species epithets is that they are formed from the 26 letters of the Latin alphabet, either upper or lower case, numbers and hyphens. Ligatures, diacritical marks, punctuation marks, subscripts, superscripts and oblique bars are not allowed. After several years of transition, culminating in the ICTV ratification vote held in February 2025, almost all virus species names are now in this format.

## Examples of virus species name changes

As described above, many different virus classification schemes have been developed over the years, and the impact that this variety has had on the format and typography of virus species names has been, to say the least, disorienting. Considering a selection of the taxonomic names associated at different times with vaccinia virus (Table 2), tobacco mosaic virus (Table 3) and Escherichia phage T2 (Table 4),^1^ it can be seen that an initial preference for a Latinized binomial diminished and then disappeared; species names acquired a variety of formats, typically not beginning with, or even including, the genus name, often becoming similar to or identical with virus names. Virus species names are now mandated to be binomial with the genus followed by a one-word freeform species epithet, a format that nearly recapitulates the very first virus species names used a century before when viruses were still novel and mysterious entities.

**Table 2.** History of taxonomic names associated with vaccinia virus

Genus	Species	Reference
*Borreliota*	*Borreliota variolae*	[45]
*Buistia*	*Buistia paschen*	[46]
*Poxvirus*	*Poxvirus officinale*	[47]
*Poxvirus*	*Poxvirus b-1*	ICTV First Report [33]
*Poxvirus*	*Vaccinia virus*	ICTV First Report [33]
*Orthopoxvirus*	*Vaccinia virus*	MSL02 (1974)
*Orthopoxvirus*	*Orthopoxvirus vaccinia*	MSL39 (2024)

**Table 3.** History of taxonomic names associated with tobacco mosaic virus*

Genus	Species	Reference
*Strongyloplasma*	*Strongyloplasma lwanowskii* Palm	[48]
(None)	*Tobacco virus I*	[5]
(None)	*Nicotiana virus I*	[29]
*Marmor*	*Marmor tabaci* var. *vulgare*	[7]
*Musivum*	*Musivum tabaci*	[49]
*Phytovirus*	*Phytovirus nicomosaicum*	[50]
*Marmor*	*Marmor tabaci*	[51]
*Protovirus*	*Protovirus tabaci*	[11]
*Tobacco mosaic virus group*	*Tobacco mosaic virus*	ICTV First Report [33]
*Tobamovirus group*	*Tobacco mosaic virus*	MSL03 (1975)
*Tobamovirus*	*Tobacco mosaic virus*	MSL13 (1993)
*Tobamovirus*	*Tobamovirus tabaci*	MSL39 (2024)

*This list of names is not exhaustive, see Table 1.1 in [52].

**Table 4.** History of taxonomic names associated with Escherichia phage T2

Genus	Species	Reference
(None)	*T2, C16 species*	[53]
*Phagovirus*	*Phagovirus T secundus*	[11]
*T-even phages*	*Coliphage T2*	ICTV First Report [33]
*T-even phage group*	*Coliphage T2*	ICTV Second Report [34]
*T4-like phages*	*Coliphage T2*	ICTV Sixth Report [38]
*T4-like viruses*	*Enterobacteria phage T4*	ICTV Seventh Report [39]
*T4-like virus*	*Enterobacteria phage T4*	MSL27 (2013)
*T4virus*	*Escherichia virus T4*	MSL30 (2016)
*Tequatrovirus*	*Escherichia virus T4*	MSL34 (2019)
*Tequatrovirus*	*Escherichia virus T2*	MSL36 (2021)
*Tequatrovirus*	*Tequatrovirus T2*	MSL37 (2022)

## Conclusions

The early assumption of virologists was that any taxonomy of viruses would employ the Latinized binomial format used elsewhere in biology. This assumption became part of the first set of Rules of Nomenclature of the ICTV. However, this expectation was not enforced or realized in practice, and species names gradually diversified in form and typography in a manner that was unhelpful to the field. The recent decision of the ICTV to mandate binomial names with a freeform species epithet has now achieved a uniform binomial format that is very close to the Latinized binomial format first envisaged almost 100 years ago [44].

Although the orthographical and typographical rules regarding the naming of the virus species taxon are now clear, this does not mean that the rules are perfect. Rule 3.11 of the current ICTV statutes states – ‘Names for taxa shall be easy to use and easy to remember. Euphonious names are preferred’. Euphony, meaning a pleasing sound, especially in speech, is arguably in the ear of the listener, but notably absent from some freeform epithets such as those employed in the current species names *Begomovirus abelmoschusbhubhaneswarense*, *Epseptimavirus phagemcphageface* and *Triavirus SA137ruMSSAST121PVL*. Mark Twain is often quoted as having said, ‘Continuous improvement is better than delayed perfection’. Whether he said it or not, this point of view is a sensible way to continue with the classification and naming of viruses.

## Footnotes

The taxonomic names are shown with the currently approved typography, although this was not always the case in the original publications

## Abbreviations

ICNV: International Committee on Nomenclature of Viruses
ICTV: International Committee on Taxonomy of Viruses
MSL: Master Species List
PCNV: Provisional Committee on Nomenclature of Viruses

## References

[1] Beijerinck MW (1898). Über ein Contagium vivum fluidum als Ursache der Fleckenkrankheit der Tabaksblätter. Verh K Akad Weterschappen Amst.

[2] Iwanowski D (1892). Über die Mosaikkrankheit der Tabakspflanze. Bull Acad Imp Sci Saint-Pétersbourg.

[3] Loeffler F, Frosch P (1898). Berichte der Kommission zur Erforschung der Maul- und Klauenseuche bei dem Institut für Infektionskrankheiten in Berlin (Teil 2). Centralbl Bakteriol Parasitenkd Infektionskrh Abt 1.

[4] Zerbini FM, Kitajima EW (2022). From Contagium vivum fluidum to *Riboviria*: a tobacco mosaic virus-centric history of virus taxonomy. Biomolecules.

[5] Johnson J (1927). The classification of plant viruses. Wis Agric Exp Stn Bull.

[6] Fawcett HS (1940). Suggestions on plant virus nomenclature as exemplified by names for citrus viruses. Science.

[7] Holmes FO (1939). Handbook of Phytopathogenic Viruses.

[8] Holmes FO, Breed RS, Hitchens AP, Murray EGD (1948). Bergey’s Manual of Determinative Bacteriology.

[9] Steinhaus EA (1949). Nomenclature and classification of insect viruses. Bacteriol Rev.

[10] Lwoff A, Horne R, Tournier P (1962). A system of viruses. Cold Spring Harb Symp Quant Biol.

[11] Lwoff A, Tournier P (1966). The classification of viruses. Annu Rev Microbiol.

[12] Gibbs AJ, Harrison BD (1968). Realistic approach to virus classification and nomenclature. Nature.

[13] Gaidamovich SY, Zhadnov VA (1966). International Congress for Microbiology.

[14] Andrewes CH (1951). Viruses and Linnaeus. Acta Pathol Microbiol Scand.

[15] Gibbs AJ, Harrison BD, Watson DH, Wildy P (1966). What’s in a virus name?. Nature.

[16] Andrewes CH (1954). Nomenclature of viruses. Nature.

[17] Baltimore D (1971). Expression of animal virus genomes. Bacteriol Rev.

[18] Goldbach RW (1986). Molecular evolution of plant RNA viruses. Annu Rev Phytopathol.

[19] Strauss JH, Strauss EG (1988). Evolution of RNA viruses. Annu Rev Microbiol.

[20] Iyer LM, Aravind L, Koonin EV (2001). Common origin of four diverse families of large eukaryotic DNA viruses. J Virol.

[21] Lauber C, Gorbalenya AE (2012). Partitioning the genetic diversity of a virus family: approach and evaluation through a case study of picornaviruses. J Virol.

[22] Moraru C, Varsani A, Kropinski AM (2020). VIRIDIC-a novel tool to calculate the intergenomic similarities of prokaryote-infecting viruses. Viruses.

[23] Bin Jang H, Bolduc B, Zablocki O, Kuhn JH, Roux S (2019). Taxonomic assignment of uncultivated prokaryotic virus genomes is enabled by gene-sharing networks. Nat Biotechnol.

[24] Mayne R, Aiewsakun P, Turner D, Adriaenssens EM, Simmonds P (2024). GRAViTy-V2: a grounded viral taxonomy application. *NAR Genom Bioinform*.

[25] Stuart DI, Oksanen HM, Abrescia NGA, Mateu MG (2024). Structure and Physics of Viruses: An Integrated Guide.

[26] Postler TS, Rubino L, Adriaenssens EM, Dutilh BE, Harrach B (2022). Guidance for creating individual and batch latinized binomial virus species names. J Gen Virol.

[27] Walker PJ, Siddell SG, Lefkowitz EJ, Mushegian AR, Adriaenssens EM (2021). Changes to virus taxonomy and to the International Code of Virus Classification and Nomenclature ratified by the International Committee on Taxonomy of Viruses (2021). Arch Virol.

[28] Johnson J (1949). Systems of virus classification and nomenclature. Tijdschrift Over Plantenziekten.

[29] Smith KM (1937). A Textbook of Plant Virus Diseases.

[30] Burnet FM (1953). Virus classification and nomenclature. Ann N Y Acad Sci.

[31] Burnet FM (1955). Principles of Animal Virology.

[32] Andrewes CH, Sneath PHA (1958). The species concept among viruses. Nature.

[33] Wildy P (1971). Classification and nomenclature of viruses. First Report of the International Committee on Nomenclature of Viruses. Monog Virol.

[34] Fenner F (1976). Classification and nomenclature of viruses. Second Report of the International Committee on Taxonomy of Viruses. Intervirology.

[35] Matthews REF (1979). Classification and nomenclature of viruses. Third Report of the International Committee on Taxonomy of Viruses. Intervirology.

[36] Matthews REF (1982). Classification and nomenclature of viruses. Fourth Report of the International Committee on Taxonomy of Viruses. Intervirology.

[37] Francki RIB, Fauquet CM, Knudson DL, Brown F (1991). Archives of Virology. Supplementum.

[38] Murphy FA, Fauquet CM, Bishop DHL, Ghabrial SA, Jarvis AW (1995). Virus Taxonomy: Sixth Report of the International Committee on Taxonomy of Viruses.

[39] Van Regenmortel MHV, Fauquet CM, Bishop DHL, Carstens EB, Estes MK (2000). Virus Taxonomy, Seventh Report of the International Committee on Taxonomy of Viruses.

[40] Fauquet CM, Mayo MA, Maniloff J, Desselberger U, Ball LA (2005). Virus Taxonomy: Eighth Report of the International Committee on Taxonomy of Viruses.

[41] King AMQ, Adams MJ, Carstens EB, Lefkowitz EJ (2012). Ninth Report of the International Committee on Taxonomy of Viruses.

[42] Van Regenmortel MHV (2001). Perspectives on binomial names of virus species. Arch Virol.

[43] Siddell SG, Walker PJ, Lefkowitz EJ, Mushegian AR, Dutilh BE (2020). Binomial nomenclature for virus species: a consultation. Arch Virol.

[44] Paillot A (1926). Sur une nouvelle maladie du noyau ou grasserie des chenilles de *P. brassicae* et un nouveau groupe de microorganismes parasites. Compt Rend.

[45] Goodpasture EW (1933). Borreliotoses: fowlpox, molluscum contagiosum, varioa-vaccinia. Science.

[46] Mackie TJ, van Rooyen CE (1937). John Brown Buist, M.D. (Edin.), B.Sc. (Edin.), F.R.C.P.Ed., F.R.S.Ed. (1846-1915): an acknowledgment of his early contributions to the bacteriology of variola and vaccinia. Edinb Med J.

[47] Fenner F, Burnet FM (1957). A short description of the poxvirus group (vaccinia and related viruses). Virology.

[48] Palm BT (1922). De mozaiekziekte van de tabak een chlamydozoonose. Medan Sumatra Deli Proefstat Bull.

[49] Valleau WD (1940). Classification and nomenclature of tobacco viruses. Phytopathology.

[50] Thornberry HH (1941). A proposed system of virus nomenclature and classification. Phytopathology.

[51] McKinney HH (1944). Genera of the plant viruses. J Wash Acad Sci.

[52] Van Regenmortel MHV (2011). Genetics and Evolution of Infectious Disease.

[53] Adams MH (1952). Classification of bacterial viruses: characteristics of the T5 species and of the T2, C16 species. J Bacteriol.

